# Purtscher-like retinopathy in a 19-year-old with maturity-onset diabetes of the young: a case report

**DOI:** 10.1186/s13256-023-03985-z

**Published:** 2023-06-19

**Authors:** Angela J. Oh, Michael Javaheri, Hamid Hosseini, Pradeep S. Prasad

**Affiliations:** 1 Department of Ophthalmology, Harbor-UCLA Medical Center, 1000 West Carson, Torrance, CA 90502 USA; 2 UCLA Stein Eye Institute, 100 Stein Plaza Driveway, Los Angeles, CA 90095 USA

**Keywords:** Case report, Diabetes, Maturity-onset diabetes of the young, Diabetic retinopathy, Purtscher’s retinopathy, 17q12 deletion

## Abstract

**Background:**

We report the first case of Purtscher-like retinopathy in a patient with 17q12 deletion-associated maturity-onset diabetes of the young.

**Case presentation:**

A 19-year-old diabetic Hispanic male with history of cataracts and toe amputations presented with sudden onset of painless bilateral vision loss for 1 week with no associated trauma. Visual acuity was counting fingers at six feet in both eyes. Dilated retinal examination revealed bilateral peripapillary cotton wool spots and intraretinal hemorrhages, and significant subretinal and intraretinal fluid on optical coherence tomography. Fluorescein angiography revealed arteriolar staining and leakage around the disc with areas of capillary nonperfusion, supporting the diagnosis of Purtscher-like retinopathy. Systemic workup revealed multiple diabetic complications including chronic osteomyelitis of multiple toes, nonhealing diabetic foot ulcers, neurogenic bladder and bowel, and bilateral lower-extremity muscular neuropathies. Genetic evaluation revealed a 17q12 deletion, which is associated with maturity-onset diabetes of the young 5. On follow-up examination, he received a single intravitreal antivascular endothelial growth factor injection in the left eye (off label) for persistent macular edema. Although his retinal edema improved, his visual acuity remained poor.

**Conclusions:**

The presentation of our patient’s multiple diabetic complications along visual symptoms suggests Purtscher-like retinopathy can be a sequela of uncontrolled diabetes. Purtscher-like retinopathy is a rare but possible consideration in diabetic patients who present with acute-onset vision loss.

## Background

Purtscher’s retinopathy is a rare cause of painless vision loss, typically seen following cranial trauma or chest compression injury [1, 2]. When associated with other systemic conditions such as pancreatitis, renal failure, and autoimmune disorders, it is identified as Purtscher-like retinopathy (PLR) [2]. Fundoscopic exam shows bilateral peripapillary cotton wool spots, retinal hemorrhages, and distinct intraretinal whitening known as Purtscher flecken [1]. There has only been one reported case of PLR associated with diabetic retinopathy, but diabetes is not a recognized risk factor [3]. We report herein a case of PLR in a patient who presented with multiple diabetic complications and was found to have a 17q12 chromosomal deletion-associated maturity-onset diabetes of the young 5 (MODY5).

## Case presentation

A 19-year-old Hispanic male with history of insulin-dependent type II diabetes presented to the emergency department with sudden onset of painless vision loss in both eyes for 1 week associated with bilateral lower-extremity weakness, inability to walk, and urinary and bowel incontinence. There was no history of trauma or family history of autoimmune, neurological, or ophthalmological conditions. Surgical history included bilateral toe amputations for diabetic foot infections 3 months prior to presentation and bilateral cataract extraction with intraocular lens implant at age 15 years. The patient had history of multiple hospitalizations including intensive care unit admissions for poorly controlled diabetes. There was no known genetic history.

**Fig. 1:**
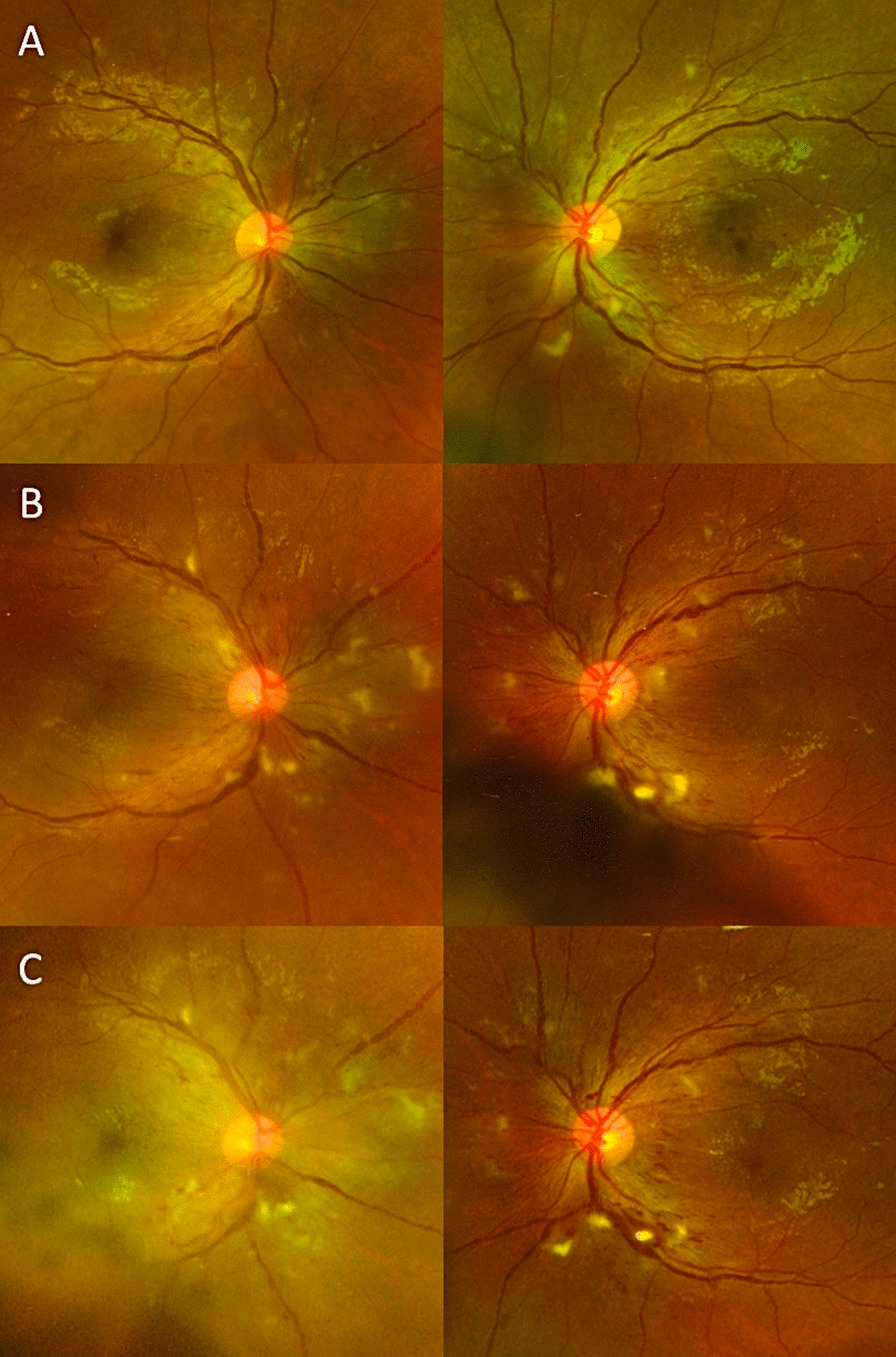
Dilated fundus photos in patient with Purtscher-like retinopathy. A 19-year-old diabetic male presented with decreased vision in both eyes for 1 week with no inciting trauma and was found to have a genetic 17q12 deletion associated with mature-onset diabetes of the young 5. **A** Diabetic retinal screening 3 months prior to initial presentation showed no evidence of diabetic retinopathy. Best corrected visual acuity was 20/20 bilaterally at this time. **B** Dilated fundus photos 2 weeks after onset of acute painless vision loss revealed multiple cotton wool spots and intraretinal hemorrhages predominantly in the peripapillary region, consistent with Purtscher-like retinopathy. There were no other signs of diabetic retinopathy beyond the peripapillary region in the posterior pole. Visual acuity was significantly decreased compared with 20/200 bilaterally. **C** One month after presentation, fundus examination showed persistent peripapillary cotton wool spots bilaterally, and visual acuity remained poor bilaterally

**Fig. 2:**
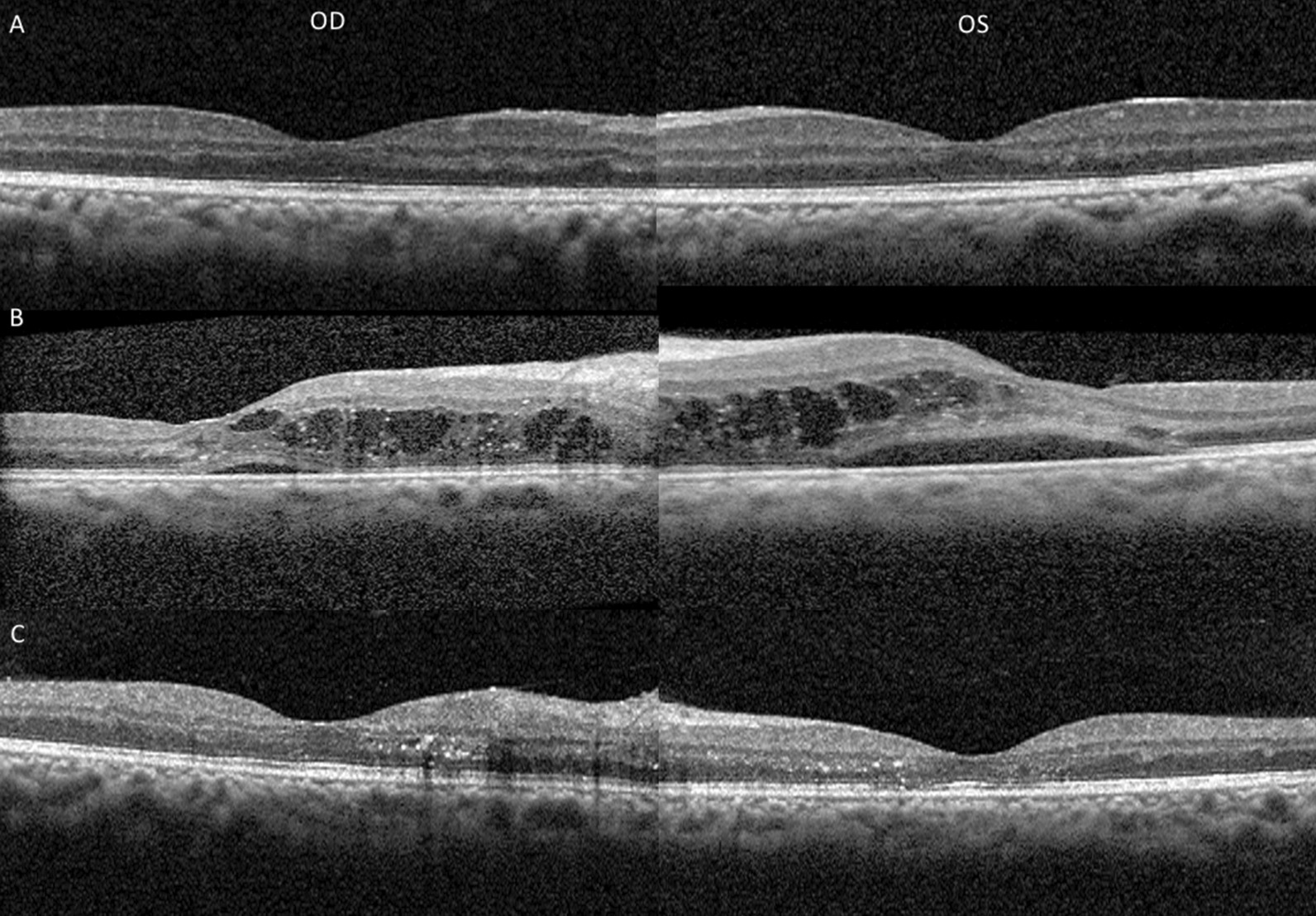
Optical coherence tomography (OCT) in a patient with Purtscher-like retinopathy. OCT photographs of both eyes in a 19-year-old diabetic male with Purtscher-like retinopathy and a genetic 17q12 deletion. **A** Baseline photographs 3 months prior to presentation showed no evidence of macular edema. **B** OCT images 2 weeks after onset of blurry vision showed large intraretinal cysts with subretinal fluid bilaterally. The patient received a single intravitreal injection of antivascular endothelial growth factor in the left eye for persistent edema 1 month after presentation. **C** OCT images 2 months after presentation showed resolution of macular edema bilaterally

On exam, best-corrected visual acuity (BCVA) was 20/200 bilaterally, significantly worse than his baseline vision of 20/20. Retinal screening from 3 months prior showed normal optic disc and vessels with few macular aneurysms and trace cotton wool spots (Fig. 1A). Pupillary exam, confrontational visual fields, ocular motility, and anterior segment exams were unremarkable. Dilated fundus examination revealed multiple peripapillary cotton wool spots and few flame hemorrhages without areas of neovascularization bilaterally (Fig. 1B). Compared with baseline spectral-domain optical coherence tomography (OCT) taken 3 months prior to presentation (Fig. 2A), OCT on presentation showed new significant areas of subretinal and intraretinal macular fluid bilaterally (Fig. 2B). Fluorescein angiography showed staining and areas of leakage from the retinal arterioles in a peripapillary distribution with multifocal filling defects and areas of capillary nonperfusion (not shown). Ophthalmic examination was consistent with Purtscher-like retinopathy.

The patient was admitted to the pediatric intensive care unit for further workup, which revealed multi-organ complications of diabetes. He had bilateral diabetic foot ulcers with recurrent toe osteomyelitis. Electromyography revealed a sensorimotor neuropathy with axonal demyelination, consistent with diabetic amyotrophy. In addition, the patient was diagnosed with neurogenic bowel and bladder, requiring daily self-catheterization and a strict bowel regimen. Labs showed a blood glucose range between 200 and 450 and HbA1c level of 5.6%. There was no evidence of cord compression, demyelination, or infection on lumbar puncture and spinal imaging. Rheumatology evaluation including sensory motor antibody, paraneoplastic autoantibody panel, and ganglioside antibody was negative. Given his young age and multiple severe downstream complications of diabetes, genetic testing was recommended. Testing revealed a 1.9-Mb deletion on 17q12 chromosome on the hepatic nuclear factor-1β gene, and the patient and his family were referred for genetic counseling.

Repeat ophthalmic examination 1 month after presentation did not show any improvement in visual acuity or resolution of peripapillary cotton wool spots (Fig. 1C). OCT showed spontaneous improvement of the macular edema in the right eye but persistent intraretinal swelling in the left. The patient received a single intravitreal injection of antivascular endothelial growth factor (1.25 mg in 0.05 mL of bevacizumab) in the left eye (off-label usage) with no known complications. This led to near resolution of the retinal edema (Fig. 2C). However, visual acuity remained poor at 20/200 in the right eye and 20/400 in the left eye.

## Discussion

Here we present the first association between 17q12 deletion-associated maturity-onset diabetes of the young 5 (MODY5) and PLR. Our patient’s presentation is unique since his acute onset of visual symptoms coincided with multi-organ diabetic disease included diabetic amyotrophy, neurogenic bowel and bladder, and recurrent osteomyelitis. Interestingly, his A1c of 5.6% was not particularly elevated. It is possible his retinal findings (cotton wool spots and macular edema) were initial features of diabetic retinopathy. However, the sudden onset of bilateral painless vision loss and minimal signs of diabetic retinopathy on retinal screening 3 months prior to presentation make this less likely. The characteristic pattern of bilateral peripapillary cotton wool spots with retinal arteriolar staining and capillary nonperfusion on fluorescein angiography as well as the lack of visual recovery are more consistent with PLR than worsened diabetic retinopathy, which does not typically present with findings solely around the optic disc.

We found one case report of PLR as the initial presentation of diabetic retinopathy, but that patient had no other systemic conditions and showed improvement in visual acuity with intravitreal injection of dexamethasone [3]. Our patient received a single intravitreal injection of antivascular endothelial growth factor in the left eye (off label usage) for persistent macular edema, and although his edema resolved, his vision did not recover. Neovascular glaucoma secondary to diabetes has also been seen with PLR, but our patient did not show evidence of iris neovascularization or increased intraocular pressure [4].

The more common systemic causes of nontraumatic PLR are acute pancreatitis, pancreatic adenocarcinoma, chronic renal failure, connective tissue disorders, and hemolytic uremic syndrome, none of which were found in our patient [2]. To the best of our knowledge, there are no reported cases of PLR in patients with MODY5 or neurogenic bowel and bladder. Our patient’s lumbosacral symptoms were diagnosed as a type of diabetic amyotrophy, an inflammatory neuropathy that led to the need for an indwelling catheter, severe physical debilitation, and weeks of therapy [5]. This condition alone is seen in only 1% of diabetics, and we found no published reports of associated ophthalmic complications. It is possible that recurrent osteomyelitis and bilateral foot infections triggered an inflammatory response that led to PLR and sudden-onset bilateral vision loss. Viral illnesses have been associated with PLR following a potentially similar vasculitic mechanism [6, 7].

This patient’s 17q12 deletion likely predisposed him to develop diabetes and its complications at a young age. This genetic finding is associated with maturity-onset diabetes of the young type 5, one of five forms of diabetes caused by a genetic mutation [8]. This chromosomal locus deletion is associated with renal cysts and diabetes syndrome as well as reduced birth weight and gout [10]. Reported ocular manifestations include bilateral punctuate cataracts, strabismus, horizontal nystagmus, hypermetropia, and coloboma [8, 9]. Our patient’s first symptom of diabetes at age 13 years (A1c of 6.9% at the time) was bilateral blurry vision from dense cataracts, and he underwent bilateral cataract surgery at age 15 years. He also had recurrent diabetic foot infections resulting in multiple toe amputations and neurogenic bowel and bladder, all devastating complications of diabetes. PLR is not recognized as a common ophthalmic complication of diabetes, but the sudden onset and severity of his visual symptoms and fundoscopic findings are unlike signs typical seen in diabetic retinopathy. The mechanism for his acute onset of multiple diabetic complications is unclear but likely point to an enhanced inflammatory response that triggered PLR.

## Conclusion

Purtscher-like retinopathy (PLR) can present as a complication of diabetes and should be considered in patients with uncontrolled disease. Our case suggests that diabetes is a risk factor for PLR.

## Data Availability

Data sharing is not applicable to this article as no datasets were generated or analyzed during the current study.

## Abbreviations

PLR: Purtscher-like retinopathy
MODY5: Maturity-onset diabetes of the young 5
BCVA: Best-corrected visual acuity
OCT: Optical coherence tomography
